# God’s Ultimatum: The Role of Delusional Guilt in Religious Psychosis

**DOI:** 10.1192/j.eurpsy.2025.1294

**Published:** 2025-08-26

**Authors:** M. Rosa, G. Santos, A. Garcia, G. da Ponte

**Affiliations:** 1 ULS Arco Ribeirinho, Lisboa, Portugal

## Abstract

**Introduction:**

Religious content often shapes the nature of psychotic delusions, particularly those involving guilt or grandiosity. Patients may interpret their faith as direct communication from a divine source, influencing both their thoughts and actions. This case report presents a 27-year-old male with a strong Evangelical background who experienced a sudden onset of manic and psychotic symptoms, including messianic delusions. He believed God gave him a divine ultimatum: either fast for 28 days or his mother would die. His response to this delusion—self-imposed starvation—led to life-threatening behavior, showcasing the impact of delusional guilt in religious psychosis.

**Objectives:**

This report aims to illustrate how delusional guilt in religious psychosis can lead to dangerous behavior, explore the role of religious context in shaping psychotic content, and discuss the clinical challenges and outcomes of pharmacological treatment in such cases.

**Methods:**

Case report; a non-systematic review was conducted by searching academic databases such as PubMed and Google Scholar, using the key-words “religion and psychosis”, “transcultural psychiatry”, “religious delusions”.

**Results:**

The patient was admitted to a psychiatric unit after attempting self-starvation as a result of his belief that God had given him a life-or-death ultimatum. His psychosis included messianic delusions, interpreting license plates as divine messages. The patient exhibited manic symptoms, such as elevated mood, hyperactivity, and pressured speech. A treatment plan with Paliperidone, titrated to 9 mg/day, and Sodium Valproate, titrated to 1000 mg/day, was initiated. After two weeks of treatment, the patient’s symptoms showed significant improvement. His delusional intensity decreased, and he began to question the validity of his beliefs. Manic symptoms also diminished. By the end of the fourth week, the patient no longer exhibited psychotic thinking, and his insight into the irrational nature of his previous beliefs had increased. He resumed normal eating habits, and the risk of further self-harm related to delusional guilt was eliminated. The patient was discharged with ongoing outpatient follow-up and maintained on the same medication regimen.

**Conclusions:**

This case illustrates the role of religious delusions in shaping dangerous behaviors, particularly when driven by delusional guilt. The patient’s belief that he was receiving divine communication leading to self-starvation highlights the clinical complexity of managing psychosis intertwined with spiritual beliefs. Cultural competence played a vital role in treatment, as understanding the patient’s religious context was essential in addressing his delusions. This case underscores the importance of individualized, culturally informed care in the management of religious psychosis.

**Disclosure of Interest:**

None Declared

